# Cost analysis of school-based intermittent screening and treatment of malaria in Kenya

**DOI:** 10.1186/1475-2875-10-273

**Published:** 2011-09-20

**Authors:** Thomas L Drake, George Okello, Kiambo Njagi, Katherine E Halliday, Matthew CH Jukes, Lindsay Mangham, Simon Brooker

**Affiliations:** 1 Faculty of Infectious and Tropical Diseases, London School of Hygiene and Tropical Medicine, London, UK; 2 Malaria Public Health & Epidemiology Group, Kenya Medical Research Institute-Wellcome Trust Collaborative Programme, Nairobi, Kenya; 3 Division of Malaria Control, Ministry of Public Health & Sanitation, Nairobi, Kenya; 4 Graduate School of Education, Harvard University, Cambridge, Massachusetts, USA; 5 Faculty of Public Health and Policy, London School of Hygiene and Tropical Medicine, London, UK

## Abstract

**Background:**

The control of malaria in schools is receiving increasing attention, but there remains currently no consensus as to the optimal intervention strategy. This paper analyses the costs of intermittent screening and treatment (IST) of malaria in schools, implemented as part of a cluster-randomized controlled trial on the Kenyan coast.

**Methods:**

Financial and economic costs were estimated using an ingredients approach whereby all resources required in the delivery of IST are quantified and valued. Sensitivity analysis was conducted to investigate how programme variation affects costs and to identify potential cost savings in the future implementation of IST.

**Results:**

The estimated financial cost of IST per child screened is US$ 6.61 (economic cost US$ 6.24). Key contributors to cost were salary costs (36%) and malaria rapid diagnostic tests (RDT) (22%). Almost half (47%) of the intervention cost comprises redeployment of existing resources including health worker time and use of hospital vehicles. Sensitivity analysis identified changes to intervention delivery that can reduce programme costs by 40%, including use of alternative RDTs and removal of supervised treatment. Cost-effectiveness is also likely to be highly sensitive to the proportion of children found to be RDT-positive.

**Conclusion:**

In the current context, school-based IST is a relatively expensive malaria intervention, but reducing the complexity of delivery can result in considerable savings in the cost of intervention.

(Costs are reported in US$ 2010).

## Background

There is a growing appreciation that malaria not only impacts on the health of infected individuals but also has broader social and economic consequences [1,2]. Recent evidence suggests that non-severe malaria can affect the cognition, attention and ultimately educational achievement of school children [3-7]. Reaching this population is most effectively achieved via the school infrastructure, and with increasing enrolment in schools by African school-age children [8], schools provide a natural access point for malaria control among this age group. Notwithstanding this potential, the optimal approach to controlling malaria in schools remains unclear [4,9]. Recent studies in Africa have demonstrated the potential of intermittent preventative treatment (IPT) - the administration of curative doses of anti-malarial treatment at predefined intervals regardless of infection status - in reducing malaria parasitaemia, clinical disease and anaemia and improving cognitive performance [3,10,11]. Moreover, modelling work demonstrates that IPT administered among school-age children can also help reduce malaria transmission in the wider community, particularly in areas of low to moderate transmission [12]. However, recent changes in national drug policies in many African countries preclude the use of sulphadoxine-pyrimethamine and amodiaquine, some of the drugs previously used in IPT, thereby limiting its potential implementation.

An alternative school-based malaria control strategy is intermittent screening and treatment (IST), using rapid diagnostic tests (RDTs) to screen and treat asymptomatic children. Recent studies in Ghana found IST in pregnant women to be equally efficacious as IPT [13] and acceptable to patients [14]. The present analysis examines the costs of IST of school children in two districts in coastal Kenya as part of an ongoing trial investigating the impact IST has on the health and education of school children [15]. The analysis explores how variation in the design of the intervention, including different component prices, affects programme cost.

## Methods

### Description of the IST trial

A factorial, cluster randomized trial is currently investigating the impact of school-based malaria control and enhanced literacy instruction on the health and educational achievement of school children in Kenya. The study design and description of the intervention are detailed elsewhere [15]. In brief, the trial is being implemented, 2010-2012, in 101 primary schools on the coast of Kenya where continuous precipitation supports moderately intense malaria transmission (predominantly *Plasmodium falciparum*). Typically, there are two seasonal peaks in malaria cases reflecting the bimodal rainfall pattern; the heaviest rainfall typically occurring between April and June and a smaller peak in October and November each year. Under nutrition, especially anaemia, is common [16]. In terms of education, the area is one of the poorest performing in Kenya, having the lowest mean national examination scores since 2005 [17].

The interventions being evaluated are (i) intermittent screening and treatment of malaria in schools by public health workers and (ii) training workshops and support for teachers to promote explicit and systematic literacy instruction (not evaluated here). The primary outcomes are educational achievement and anaemia. Secondary outcomes include malaria parasitaemia, school attendance, sustained attention and other cognition abilities. Randomly selected children from classes 1 and 5 are included in the evaluation. Baseline health and education surveys were conducted in intervention and control schools between January and March 2010, with 12 and 24-month follow-up surveys scheduled.

### Intermittent screening and treatment (IST)

Each school term, all children are tested for malaria using a RDT. The RDT used is a ParaCheck-*Pf *device (Orchid Biomedical Systems, Goa, India) which is able to detect *P. falciparum *and other (unspecified) *Plasmodium *species. Children (with or without malaria symptoms) found to be RDT-positive are treated with artemether-lumefantrine, AL (Coartem^®^, Novartis), an artemisinin-based combination therapy. Testing and treatment is administered by district health workers and supported by the Division of Malaria Control (DoMC), Ministry of Public Health and Sanitation (MoPHS).

On day 1, children are screened by a laboratory technician using a RDT. Those children found to be RDT-positive are given milk and bread and then given the first dose of AL. Parents or older siblings of children are called and a nurse explains that their child is infected with malaria parasites and requires treatment (assuming they are not already taking medication). The parents/older siblings are given the second dose of AL and told that this should be taken in the evening with food. On day 2, the nurse returns to the school, gives the third AL dose to children and provides the parent/older sibling with the fourth dose. Children absent from school are followed up at their home and provided with the doses. On day 3, the procedures are the same as day 2. During follow-up visits nurses monitor for potential side effects of treatment.

### Costing

The analysis is undertaken from the perspective of the Government of Kenya, as a public service provider. Only costs to the provider are included as costs to the patient of accessing the intervention are likely to be low since it is delivered in schools and there is no fee to receive the intervention. The costs of accessing IPT have previously been considered negligible on the same basis [18,19]. The comparison (null) for this evaluation is no intervention. The total economic cost is calculated based on an initial 5 year programme implementation. The decreased value placed on future costs and annualization of capital costs is calculated using a 3% discount rate, in line with WHO recommendations [20]. The financial costs are the unadjusted funds required to finance the intervention and the economic cost reflects the total resource burden, taking into account the value of donated goods or unpaid workers.

Programme costing was guided by a three-step process: resource identification, resource measurement and resource valuation. In this process, relevant unit costs were collected according to an ingredients based approach [21], the quantity or usage of each ingredient was determined and combined with cost information to produce a monetary valuation of total resources used, or economic cost. Costs were separated into those that required new funds, such as the purchase of additional RDTs and antimalarials, and those that involved the redeployment of existing resources, including use of health workers who would otherwise have duties at the local health facility.

Data collection was undertaken in 2010, with unit costs established from the project accounting system and from interviews with purchasing officers. Where information was unavailable or unrepresentative, unit costs were sourced from the Ministry of Public Health and Sanitation (MoPHS) or wholesale market prices. Ingredient usage was established from direct observation of the intervention, interviews with trial co-ordinators, from health worker time sheets and driver mileage survey. The majority of costs were collected in Kenyan Shillings (KES) and then converted to US$ using the average exchange rate from the preceding 12 months (01.08.09 to 31.07.10): US$ 1 = KES 79.9 [22]. Costs derived from other years were inflated or deflated to 2010, using a compound inflation factor based on the year by year consumer price index [23]. The World Health Organization CHOosing Interventions that are Cost-Effective (WHO CHOICE) [24] was used to determine the country specific item lifespan of capital items: vehicle 8 years, personal computer 10 years, printer 10 years. Costs relating to activities solely for research purposes were excluded. To account for resource waste through faulty goods, mishandling or accidents, a wastage factor of 10% was applied to all relevant items.

Intervention costs were grouped by resource type including: personnel; transport; field equipment; and health facility costs. In addition, costs were broken down by the various components or activities of the intervention including: community sensitisation; screening day; treatment days; administration; training and monitoring. Community sensitisation involves a meeting with parents and teachers at every school to describe the intervention and answer questions. This occurs once and comprises the set-up costs of the intervention, thus costs were annualized across the five-year programme. Screening day is the first day of the intervention, children are screened and treatment is started. Days two and three are treatment days where a nurse returns to the school to supervise the morning treatment and deliver the evening dose. Administration includes coordinator time, office use and the cost of distributing significant extra quantities of RDTs and anti-malarials to district hospitals. Training on the intervention delivery and a refresher of relevant clinical practice is given to all staff at every screening round. Monitoring of intervention delivery is undertaken by supervising health officers joining two intervention teams for observation at every round. A summary of intervention components is found in Table 1. Activity cost is cross-tabulated against resource category to provide a concise but detailed account of cost distribution. Unit costs are provided in the supplementary information (Additional file 1).

**Table 1:** Components of the intermittent screening and treatment in school children in coastal Kenya

**1. Community sensitisation**	Sensitization consisted of a meeting with parents and teachers at every school to describe the intervention and answer questions. It occurs once and comprises the set-up costs of the intervention, thus costs are annualized across the five-year programme.
**2. Training**	A half-day training on the intervention delivery and a refresher of relevant clinical practice is given to all staff at every screening round.
**3. Screening day**	A mobile health team travels to the school. Children are screened by a laboratory technician using a RDT and those found to be RDT-positive are given milk and bread and the first dose of treatment. The evening dose is given to the child or if the child is too young to take responsibility the parents or older sibling are called.
**4. Treatment Follow-up**	On days two and three a nurse returns to the school to supervise the morning treatment dose and deliver the evening dose.
**5. Monitoring**	Supervising health officers join two intervention teams for observation at each round.
**6. Administration**	This includes coordinator time, office use and the cost of distributing significant extra quantities of RDTs and anti-malarials to district hospitals.

### Sensitivity analysis

Univariate sensitivity analysis was conducted to determine how sensitive costs are to variation in input parameters, including commodity prices, the design of the delivery strategy, and evaluation methodology. Results are displayed graphically using a tornado diagram. For anti-malarials and RDTs, the highest and lowest prices of equivalents available in Kenya were chosen. Other variables examined include salary levels (± 20%); discount rate (0%, 5%), and wastage factor (0%, 20%). To investigate the marginal cost of supervising treatment, health worker attendance on days 2 and 3 were removed, with parents/older siblings being given a full treatment course and instructions on how to administer treatment on the screening day. The second intervention change was the removal of technicians from the screening teams, with nurses from local health facilities carrying out RDT testing. The current estimates for time spent at schools includes preparation of blood slides and collection of research information. For the sensitivity analysis is it estimated that nurses could carry implement IST without a technician under non-research conditions.

A final parameter investigated was the prevalence of *Plasmodium falciparum *in the target population, a factor that will determine the quantity of anti-malarial treatments used. Simulations using bespoke scripting in Microsoft Excel (2007) were performed, whereby variation in prevalence of infection was simulated through repeated sampling of programme cost at random prevalences. One thousand repetitions were performed in order to cover the full prevalence range. The cost per child screened and cost per child treated are plotted to present variation in programme cost and cost-effectiveness (the number of children treated is a proxy measure of effect).

Ethical and scientific approval for the present study is provided as part of the wider trial by the Kenya Medical Research Institute and National Ethics Review Committee (SSC No. 1543), the London School of Hygiene and Tropical Medicine (LSHTM) Ethics Committee (5503), and the Harvard University Committee on the Use of Human Subjects in Research (F17578-101). Sponsorship and insurance is provided by the LSHTM's Clinical Trials Sub-Committee (QA225).

## Results

The total financial cost of providing a five-year programme of malaria screening and treatment to 3,685 children is estimated to be US$ 365,104 or US$ 6.61 per child screened. The economic costs of the programme are US$ 69,062 per year, US$ 6.24 per child screened or US$ 18.72 per child per year. Table 2 provides a breakdown of financial and economic costs. The largest single contributors to cost are salaries (36%) and RDTs (22%). Almost half (47%) of the intervention cost comprises redeployment of existing resources including health worker time and use of hospital vehicles. The new funds required are largely due to RDTs and other consumables, their distribution to local facilities and staff per diems. Table 3 presents the resource costs cross-tabulated against the intervention activities and shows that the majority of the costs are spent on screening (52%), then treatment follow-up (21%) and intervention administration (20%). Data from the health worker time surveys indicates that daily travel to and from the schools during screening took on average 3 hours 20 minutes or 47% of total time. Undertaking the screening and providing treatment took 3 hours 16 minutes (45%), with preparation in the schools taking 36 minutes (8%).

**Table 2:** Financial and economic costs of malaria intermittent screening and treatment in schools in coastal Kenya by resource category (US$ 2010)

	Financial Cost^1^			Annual Economic Cost	Economic cost per child screened	**Cost Profile (%)**^**6**^
				
Resource	New funds	Existing resources	Total			
**Personnel:**						
Salaries	-	132,516	132,516	25,077	2.27	36
Per Diems	22,852	-	22,852	4,357	0.39	6
	
	**22,852**	**132,516**	**155,368**	**29,434**	**2.66**	**43**
**Transport:**						
Vehicle	-	17,387	17,387	3,292	0.30	5
Fuel	11,771	-	11,771	2,229	0.20	3
Servicing	-	16,884	16,884	3,197	0.29	5
Distribution^2^	33,104	-	33,104	6,246	0.57	9
	
	**44,875**	**34,271**	**79,146**	**14,965**	**1.35**	**22**
**Facility:**						
Rent^3^	-	5,016	5,016	957	0.09	1
Other^4^	2,761	-	2,761	534	0.05	1
	
	**2,761**	**5,016**	**7,777**	**1,490**	**0.13**	**2**
**Field Equipment:**						
RDTs	80,650	-	80,650	15,217	1.38	22
Anti-malarials	9,919	-	9,919	1,872	0.17	3
Other^5^	32,243	-	32,243	6,084	0.55	9
	
	**122,813**	**-**	**122,813**	**23,173**	**2.10**	**34**
**TOTAL**	**193,301**	**171,803**	**365,104**	**69,062**	**6.24**	**100**

**%**	**53**	**47**				
					

^1 ^Financial costs are the undiscounted direct monetary costs for the programme over five years.

^2 ^Cost of transporting extra RDTs and anti-malarials to the district hospital

^3 ^Includes utilities and furniture.

^4 ^Includes office consumables and computer equipment.

^5 ^Includes blood lancets, cotton wool, gauze roll, gloves, paper towels, disinfectant dispenser, thermometer, biscuit packs, milk cartons, bottled water, paracetamol, pencils, erasers, sharpeners, masking tape, garbage bag, marker pens, scissors, dust bin, triple timers, weighing scales and mobile phone credit.

^6 ^Applies to both financial and economic costs.

**Table 3:** The costs of malaria intermittent screening and treatment in schools in coastal Kenya by resource category and intervention activity (US$ 2010)

	Resource					
Activity	Personnel	Transport	Facility	Field Equipment	TOTAL	%
Sensitisation	872	231	166	-	**1,270**	2
Training	943	-	44	17	**1,003**	1
Screening	12,642	2994	-	20,399	**36,035**	52
Follow-Up	6,317	5,494	-	2,757	**14,568**	21
Monitoring	2,126	-	132	-	**2,258**	3
Administration	6,535	6,246	1,148	-	**13,929**	20

**TOTAL**	**29,434**	**14,965**	**1,490**	**23,173**	**69,062**	**100**
%	43	22	2	34	**100**	

### Sensitivity analysis

**Figure 1:**
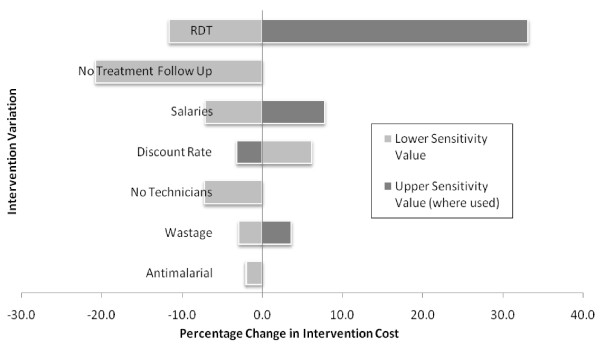
**Tornado diagram of the percentage change in the cost of intermittent screening in Kenyan schools in relation to variation in component costs**.

The parameters included in the sensitivity analysis and the change in cost per child screened are detailed in Table 4 and displayed graphically in Figure 1. Choice of RDT had a large impact on overall costs (12% reduction or 33% increase), whereas drug choice had negligible impact. The biggest cost saving was removing the treatment follow-up (21%), whilst not including technicians in the screening teams reduced costs by 7%. Other variations altered costs by less than 10%. This analysis identifies three intervention alterations which together may be expected to reduce total cost by 40% without significantly altering the fidelity of delivery: (i) using a cheaper RDT brand; (ii) removing directly observed treatment follow-up; and (iii) removing technicians from health teams and allowing nurses to carry out RDTs.

**Table 4:** Sensitivity analysis of the costs of malaria intermittent screening and treatment in schools in coastal Kenya

Parameter	Parameter Baseline Value	Variation and Justification	Cost Per Child Screened (% Change)	
		Baseline Result:	$6.24	
			Lower Value	Upper Value
RDT	Paracheck: $1.32	First Response: $0.61 NOW Malaria: $3.21 The cheapest and most expensive high performing alternatives considered by the Kenyan government.	$5.52 (-12%)	$8.31 (+33%)
Anti-malarial	AL: $0.31 - 1.23 depending on child weight	AQ + SP: $0.125 DP: $0.741 Dihydroartemisinin Piperaquine (DP) is an alternative ACT while Amodiaquine Sulphadoxine-Pyrimethamine (AQ + SP) is a cheap alternative that might be used in an area where SP is still effective.	$6.12 (-2%)	$6.24 (< 1%)
Treatment Follow Up	Treatment follow up carried out by nurses as described	Unsupervised treatment has been shown to be similarly efficacious [37,38] and national guidelines permit unsupervised treatment [39]. Alternative treatment may also reduce follow up requirements.	$4.95 (-21%)	-
Health Team Personnel	Technicians used by trial to carry out RDT and blood slide.	Nurses implement IST without technicians. Personnel may be reduced by removing research tasks such as taking blood slides and anthropometry.	$5.79 (-7%)	-
Salaries	Midpoint of relevant pay scales.	± 20% Salaries are likely to vary by region or over time	$5.80 (-7%)	$6.73 (+8%)
Discount Rate	3% Recommended by WHO [20]	0% and 5% 0% reflects un adjusted programme costs. Some argue that time preferences for delay of costs are not necessarily rational and should not be included in decision-making. 5% represents a greater time preference, argued by some to be more relevant to developing country contexts.	$6.63 (+6%)	$6.04 (-3%)
Wastage	10%	0% and 20% No empirical evidence. Based on literature precedent.	$6.06 (-3%)	$6.47 (+4%)

**Figure 2:**
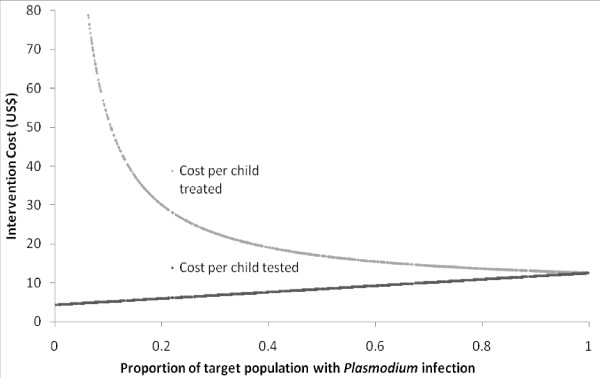
**The relationship between the cost of school-based intermittent screening and the prevalence of *Plasmodium falciparum *in school children**.

Figure 2 shows the relationship between the prevalence of *P. falciparum *infection (as based on RDT results) and the cost per child screened and cost per RDT-positive child treated. As RDT-positivity increases, the cost per child screened increases in a linear fashion since more anti-malarials are required. However, as prevalence of infection decreases the cost per child treated rises exponentially.

## Discussion

The current analysis estimates that the financial cost of IST is US$ 6.61 per child screened. The economic cost is US$ 6.24 although the difference is largely due to discounting of costs incurred in the future. The largest cost components were RDTs and salaries. Almost half (47%) of the costs comprised of the redeployment of existing resources including health worker time and use of hospital vehicles. While this may reduce the new funds required to finance the intervention it also highlights the potential for additional strain to be placed on existing resources, depending on the current working capacity. Sensitivity analysis highlighted that changes to the delivery strategy, such as using different RDTs, removing treatment follow-up and only including nurses in the screening team, can reduce overall costs by 40%, increasing the affordability of the intervention.

Although IST is principally a strategy of treatment rather than prevention of malaria, it can be compared to preventive interventions such as IPT in that it seeks to prevent malaria associated anaemia and its consequences in school children [3,10]. Studies in western Kenya found the yearly cost of school-based IPT to be US$ 3.17 per child [18] (Unless otherwise stated costs are adjusted to US$ 2010 using national CPI). Notwithstanding inflationary differences, the yearly cost of three rounds of IST at US$ 18.72 per child is considerably higher than IPT. It should be noted however, that the low cost of IPT hinges on the ability to use SP or amodiaquine, which is no longer possible in Kenya due to changes in national drug policy withdrawing SP and amodiaquine monotherapy. The ACT dihydroartemisinin-piperaquine (DP) has been considered as an alternative for IPT in school children [11], but would increase IPT costs [18]. The cost of IPT for children (IPTc) aged between 1 and 5 years in Ghana is estimated to be US$ 11.38 to US$ 14.79 [19] while a comparison of strategies for delivering IPTc in the Gambia estimated costs of US$ 3.85 and US$ 1.81 if delivered by village health workers or reproductive and child health trekking teams respectively [25]. This illustrates the opportunities for cost reduction depending on delivery strategy and highlights the potential difference in cost between settings. Multi-site studies of IPT for infants (IPTi) and for insecticide-treated bed net (ITN) distribution found respective yearly costs to be in the region of US$ 1.36 to US$ 4.03 per child (US$ 2007) [26] and US$ 1.38 and US$ 1.90 per child (US$ 2005) [27]. Studies are also beginning to consider screening strategies for targeting asymptomatic malaria infection in the wider community [28,29], although estimates of cost are not yet available.

The sensitivity analysis provides an indication of how the costs of IST may be reduced in the future. A variety of malaria RDTs are currently available, but have varying diagnostic performance and any cost savings in using different RDTs or an alternative diagnostic tool will need to be balanced against performance. In the current study, the cheaper RDT considered in the sensitivity analysis performs slightly better than the trial RDT according to studies by the World Health Organization [30]. RDT diagnosis performs similarly to or even better than routine microscopy in health centres [31], although a lower mean parasite density in asymptomatic infection may affect diagnostic performance. A study of school children with and without fever in Benin found that while PCR diagnosis led to a considerable increase in identified *Plasmodium *infection compared to microscopy or RDT, there was little or no clinical benefit in treating sub-microscopic infection [32]. Moreover, the costs of screening programmes using microscopy or PCR diagnosis would likely be prohibitively expensive. While cost-effectiveness of RDTs compared to microscopy in health centres depends on the local setting [33-36], the logistical disadvantage of microscopy as a mobile diagnostic tool means it is unlikely to be cost-effective for school-based IST.

The change to unsupervised treatment is justified on (i) findings from previous clinical trials indicating that supervised treatment and unsupervised treatment are equivalent in terms of efficacy [37,38] and (ii) that national guidelines permit unsupervised treatment [39]. In addition to the likely reduction in health worker personnel time on implementation, there may be scope for teachers to assist or even lead screening. Studies in Zambia and the Democratic Republic of Congo have shown that RDTs can be used effectively by non-medical personnel [40-42]. The wider regional or national roll out of IST may result in reduced RDT cost from bulk purchasing and improved efficiency from familiarity with the intervention. However, as coverage expands to include those in more remote areas, increased transport and therefore personnel time may increase marginal cost. In addition, integration with other school-based or community health programmes provides further potential cost reduction through economies of scope. For example, school health programmes currently provide children with de-worming and micronutrient supplementation [43]. The UNICEF led Child Health Day programme can cost-effectively deliver multiple health services to younger children [44] and bed net distribution has been successfully integrated with routine or campaign vaccination strategies [45]. IST implementation would need to be tailored to the epidemiological context and take into account infrastructural capacity and local geography. The cost, and ultimately cost-effectiveness, will crucially depend on the prevalence of *Plasmodium *infection, with overall costs decreasing with decreasing prevalence but costs per child treated increasing exponentially. However, this does not incorporate potentially crucial cost savings associated with reducing malaria transmission. That is, this strategy may indeed be cost-effective at low to moderate prevalence if elimination, or even reduction in transmission, is achieved.

There are certain limitations associated with economic evaluations of randomized trials. In this case, the intervention is designed with the trial objectives in mind and not maximisation of cost-effectiveness, for example only two classes per school were screened. In the base case scenario all research only costs were removed but intervention alterations were not modelled. The intention is to maximize the level certainty in the base case estimate, providing a solid estimate of cost from which to model variation in setting or intervention design. Findings can be generalized for settings with similar social, economic and epidemiological conditions, such as Western or Nyanza provinces [16]. Although specific figures will change, options for cost reduction, issues around scale-up and discussion of consequences are likely to remain relevant. A degree of socio-economic, epidemiological and environmental consistency is assumed and the analysis does not account for significant exogenous variation. In particular, it is assumed that the prevalence of malaria remains constant year on year. It is noted that this is unlikely to be the case in the study setting since transmission is currently in decline [46].

In order to set the context of IST cost, the likely consequences of IST are identified through consideration of the trial outcomes and a review of the relevant literature (Table 5). The aetiology of anaemia and its interaction with malaria is complex but treating asymptomatic *Plasmodium *infection using IPT has been found to reduce anaemia by 48% in school children in western Kenya [3] and 47-56% in children under five years of age in Mali and Burkina Faso [47,48]. Although IST targets asymptomatic infection there is likely to be an impact on the incidence of clinical malaria through the reduction of superinfection [49], whereby infection from an initial mosquito bite has not cleared before a second infectious bite occurs. There may also be a prophylactic effect of AL against new infection [50], an effect likely to vary according to drug choice. In addition to the benefits for individuals receiving IST, there may be effects on the wider community. In endemic regions, individuals with chronic asymptomatic infection, so-called asymptomatic carriers, are thought to represent a significant reservoir of *Plasmodium *transmission and treatment of such carriers can help reduce overall transmission in the community [51-53]. Mathematical modelling of community-based screening and treatment and, separately, of IPT in school children highlight the potential for reducing malaria transmission in this way [12,54]. Further to these effects, school-level estimates of infection prevalence derived from IST can help inform surveillance of malaria transmission [55], a key resource in disease control. Finally, by targeting asymptomatic infection, IST exposes a new section of the parasite population to an anti-malarial resistance selection pressure. Yeung *et al *describe the benefit of asymptomatic *Plasmodium *infections in suppressing the spread of drug resistance [56].

**Table 5:** The hypothesized consequences of malaria intermittent screening and treatment in schools in coastal Kenya

Consequences	Justification of Consequence	
Health		
Reduced anaemia	48% reduction of moderate anaemia was found from IPT in Kenyan school children and improved haemoglobin in Ugandan children using IPT.	[3,11]
Reduced clinical malaria	Reductions of between 42% and 67% reported by seasonally targeted IPT trials in schools in Mali.	[10,61]
Reduced malaria transmission	Modelling studies of community based IST and school based IPT suggests potential for considerable impact on transmission of malaria due to treatment of asymptomatic disease reservoir	[12,54]
Surveillance data	Potential of school malaria surveys for community surveillance of malaria. Early detection systems for malaria in the Kenyan highlands were found to cost US$15,512 per district.	[16,55,62,63] [64]
Drug resistance	Modelling studies highlight the increased resistant parasite selection pressure due to treatment of asymptomatic infection.	[56,65,66]
**Non-Health**		

Improved educational achievement	Improvement in attendance, cognitive ability and educational attainment found from IPT or chemoprophylaxis in school children.	[3,5,7]
Cost reductions	Local household cost burden reduced by US$ 2.52 per malaria episode.	[57]

With regard to impact on malaria transmission, Kern et al [54] found that intermittent community screening campaigns using RDTs followed by treatment of asymptomatic carriers has greatest impact in areas of high transmission but that the rate of infection returned to its normal level in the subsequent year, unless the intervention was repeated, highlighting the potential of community-based IST in reducing malaria transmission. In areas of low transmission, the reduction in infection was sustained for over three years following a single round of intervention. The impact of screening and treating only school children on the overall level of transmission in the wider community remains to be established, but Aguas et al find that IPT in school children has the potential to reduce transmission particularly in areas of low or moderate endemicity [12]. Kern et al also found that screening campaigns scheduled in close succession (monthly intervals) at the start of the dry season had the greatest impact on the success of the intervention. Such findings have relevance for the optimal delivery of school-based IST.

In addition to the impact on health and malaria control, school-based IST has the potential to improve education and reduce household costs. Malaria is considered to impact children's educational achievement through reduced school attendance due to illness and through impaired concentration and cognition [3-7]. Recent work by Chuma *et al *estimated the average cost to the household per episode of malaria in the study districts, Kwale and Msambweni, to be US$ 2.52 [57] [Jane Chuma, personal communication]. The above breadth of potential consequences of school-based IST necessitates that a broad perspective to economic evaluation is adopted. A possible approach is cost-consequence analysis (CCA), a framework which presents, but does not aggregate, multiple outcomes. Coast *et al *[58] and Williams *et al *[59] support CCA as a clear and comprehensive approach to economic evaluation whilst Weatherley *et al *[60] recommend a CCA framework be used in the evaluation of intersectoral costs and consequences.

## Conclusions

This analysis shows that in the current setting IST in schools is a relatively expensive intervention, primarily due to the RDT costs and the follow-up visits to observe treatment on day 2 and 3. However, many of the costs represent redeployment of existing resources and future alteration in the design of the intervention can reduce costs by 40%. Future research will evaluate impact of IST in schools and how this might vary in different transmission and operational settings.

## Competing interests

The authors declare that they have no competing interests.

## Authors' contributions

TD led the data collection, data analysis and developed the manuscript. GO, KN, KEH were responsible for fieldwork supervision and contributed to the final manuscript. MCHJ and LM contributed to study design, interpretation and scientific guidance. SB was responsible for the overall project management, study design, scientific guidance and writing of the manuscript. All authors read and approved the final manuscript.

## Supplementary Material

Additional file 1 **Unit Costs**. A list of ingredient unit costs and relevant data collected for this evaluation. Click here for file

## References

[B1] Jones COH Williams HA The social burden of malaria: what are we measuring? Am J Trop Med Hyg 2004 71 156 161 15331832

[B2] Sachs J Malaney P The economic and social burden of malaria Nature 2002 415 680 685 10.1038/415680a 11832956

[B3] Clarke SE Jukes MCH Njagi JK Khasakhala L Cundill B Otido J Crudder C Estambale BBA Brooker S Effect of intermittent preventive treatment of malaria on health and education in schoolchildren: a cluster-randomised, double-blind, placebo-controlled trial Lancet 2008 372 127 138 10.1016/S0140-6736(08)61034-X 18620950 PMC2495044

[B4] Brooker S Malaria control in schools: a toolkit of effective education sector responses to malaria in Afria World Bank, Washington DC, USA and Partnership for Child Development 2009 1 60

[B5] Fernando D de Silva D Carter R Mendis KN Wickremasinghe R A randomized, double-blind, placebo-controlled, clinical trial of the impact of malaria prevention on the educational attainment of school children Am J Trop Med Hyg 2006 74 386 393 16525095

[B6] Fernando SD Rodrigo C Rajapakse S The 'hidden' burden of malaria: cognitive impairment following infection Malar J 2010 9 366 10.1186/1475-2875-9-366 21171998 PMC3018393

[B7] Jukes MCH Pinder M Grigorenko EL Smith HB Walraven G Bariau EM Sternberg RJ Drake LJ Milligan P Cheung YB Greenwood BM Bundy DAP Long-term impact of malaria chemoprophylaxis on cognitive abilities and educational attainment: follow-up of a controlled trial PLoS clinical trials 2006 1 e19 10.1371/journal.pctr.0010019 17013430 PMC1851720

[B8] United Nations The Millenium Development Goals Report Book The Millenium Development Goals Report 2010 (Editor ed.^eds.). City

[B9] Brooker S Clarke S Snow RW Bundy DA Malaria in African schoolchildren: options for control Trans R Soc Trop Med Hyg 2008 102 304 305 10.1016/j.trstmh.2008.01.010 18313705 PMC2653942

[B10] Barger B Maiga H Traore OB Tekete M Tembine I Dara A Traore ZI Gantt S Doumbo OK Djimde AA Intermittent preventive treatment using artemisinin-based combination therapy reduces malaria morbidity among school-aged children in Mali Trop Med Int Health 2009 14 784 791 10.1111/j.1365-3156.2009.02294.x 19497079 PMC3038653

[B11] Nankabirwa J Cundill B Clarke S Kabatereine N Rosenthal PJ Dorsey G Brooker S Staedke SG Efficacy, safety, and tolerability of three regimens for prevention of malaria: a randomized, placebo-controlled trial in Ugandan schoolchildren PLoS ONE 2010 5 e13438 10.1371/journal.pone.0013438 20976051 PMC2957410

[B12] Aguas R Lourenço JML Gomes MGM White LJ The impact of IPTi and IPTc interventions on malaria clinical burden - in silico perspectives PLoS ONE 2009 4 e6627 10.1371/journal.pone.0006627 19675675 PMC2722080

[B13] Tagbor H Bruce J Agbo M Greenwood BM Chandramohan D Intermittent screening and treatment versus intermittent preventive treatment of malaria in pregnancy: a randomised controlled non-inferiority trial PLoS ONE 2010 5 e14425 10.1371/journal.pone.0014425 21203389 PMC3010999

[B14] Smith LA Jones C Adjei RO Antwi GD Afrah NA Greenwood B Chandramohan D Tagbor H Webster J Intermittent screening and treatment versus intermittent preventive treatment of malaria in pregnancy: user acceptability Malar J 2010 9 18 10.1186/1475-2875-9-18 20074372 PMC2817700

[B15] Brooker S Okello G Njagi K Dubeck MM Halliday KE Inyega H Jukes MCH Improving educational achievement and anaemia of school children: design of a cluster randomised trial of school-based malaria prevention and enhanced literacy instruction in Kenya Trials 2010 11 93 10.1186/1745-6215-11-93 20929566 PMC2959045

[B16] Gitonga CW Karanja PN Kihara J Mwanje M Juma E Snow RW Noor AM Brooker S Implementing school malaria surveys in Kenya: towards a national surveillance system Malar J 2010 9 306 10.1186/1475-2875-9-306 21034492 PMC2984573

[B17] RTI International Early Grade Reading Kenya: Baseline Assessment 2008 1 54

[B18] Temperley M Mueller DH Njagi JK Akhwale W Clarke SE Jukes MCH Estambale BBA Brooker S Costs and cost-effectiveness of delivering intermittent preventive treatment through schools in western Kenya Malar J 2008 7 196 10.1186/1475-2875-7-196 18826594 PMC2564968

[B19] Conteh L Patouillard E Kweku M Legood R Greenwood B Chandramohan D Cost effectiveness of seasonal intermittent preventive treatment using amodiaquine & artesunate or sulphadoxine-pyrimethamine in Ghanaian children PLoS ONE 2010 5 10.1371/journal.pone.0012223 PMC2923188 20808923

[B20] T Tan-Torres Ededjer CJLM Making choices in health: WHO guide to cost-effectiveness analysis 2003

[B21] Drummond M Sculpher M Torrance G O'Brien B Stoddart G Methods for the Economic Evaluation of Health Care Programmes 2005 Third Oxford University Press

[B22] Historical Exchange Rates (OANDA) http://www.oanda.com/currency/historical-rates

[B23] Kenya Inflation rate (consumer prices) http://www.indexmundi.com/kenya/inflation_rate_(consumer_prices).html

[B24] World Health Organization WHO CHOICE (CHOosing Interventions that are Cost-Effective): Useful Life

[B25] Bojang KA Akor F Conteh L Webb E Bittaye O Conway DJ Jasseh M Wiseman V Milligan PJ Greenwood B Two Strategies for the Delivery of IPTc in an Area of Seasonal Malaria Transmission in The Gambia: A Randomised Controlled Trial PLoS Med 2011 8 e1000409 10.1371/journal.pmed.1000409 21304921 PMC3032548

[B26] Conteh L Sicuri E Manzi F Hutton G Obonyo B Tediosi F Biao P Masika P Matovu F Otieno P Gosling RD Hamel M Odhiambo FO Grobusch MP Kremsner PG Chandramohan D Aponte JJ Egan A Schellenberg D Macete E Slutsker L Newman RD Alonso P Menéndez C Tanner M The cost-effectiveness of intermittent preventive treatment for malaria in infants in Sub-Saharan Africa PLoS ONE 2010 5 e10313 10.1371/journal.pone.0010313 20559558 PMC2886103

[B27] Yukich JO Lengeler C Tediosi F Brown N Mulligan J-A Chavasse D Stevens W Justino J Conteh L Maharaj R Erskine M Mueller DH Wiseman V Ghebremeskel T Zerom M Goodman C McGuire D Urrutia JM Sakho F Hanson K Sharp B Costs and consequences of large-scale vector control for malaria Malar J 2008 7 258 10.1186/1475-2875-7-258 19091114 PMC2625363

[B28] Stresman GH Kamanga A Moono P Hamapumbu H Mharakurwa S Kobayashi T Moss WJ Shiff C A method of active case detection to target reservoirs of asymptomatic malaria and gametocyte carriers in a rural area in Southern Province, Zambia Malar J 2010 9 265 20920328 10.1186/1475-2875-9-265 PMC2959066

[B29] Ogutu B Tiono AB Makanga M Premji Z Gbadoé AD Ubben D Marrast AC Gaye O Treatment of asymptomatic carriers with artemether-lumefantrine: an opportunity to reduce the burden of malaria? Malar J 2010 9 30 10.1186/1475-2875-9-30 20096111 PMC2824802

[B30] World Health Organization Malaria rapid diagnostic test performance 2009 1 34

[B31] de Oliveira AM Skarbinski J Ouma PO Kariuki S Barnwell JW Otieno K Onyona P Causer LM Laserson KF Akhwale WS Slutsker L Hamel M Performance of malaria rapid diagnostic tests as part of routine malaria case management in Kenya Am J Trop Med Hyg 2009 80 470 474 19270300

[B32] Faucher J-F Aubouy A Béhéton T Makoutode P Abiou G Doritchamou J Houzé P Ouendo E Deloron P Cot M What would PCR assessment change in the management of fevers in a malaria endemic area? A school-based study in Benin in children with and without fever Malar J 2010 9 224 10.1186/1475-2875-9-224 20691048 PMC2925366

[B33] Shillcutt S Morel C Goodman C Coleman P Bell D Whitty CJM Mills A Cost-effectiveness of malaria diagnostic methods in sub-Saharan Africa in an era of combination therapy Bull World Health Organ 2008 86 101 110 10.2471/BLT.07.042259 18297164 PMC2647374

[B34] Lubell Y Hopkins H Whitty CJM Staedke SG Mills A An interactive model for the assessment of the economic costs and benefits of different rapid diagnostic tests for malaria Malar J 2008 7 21 10.1186/1475-2875-7-21 18226224 PMC2266929

[B35] Yukich J D'Acremont V Kahama J Swai N Lengeler C Cost savings with rapid diagnostic tests for malaria in low-transmission areas: evidence from Dar es Salaam, Tanzania Am J Trop Med Hyg 2010 83 61 68 10.4269/ajtmh.2010.09-0632 20595479 PMC2912577

[B36] Uzochukwu BSC Obikeze EN Onwujekwe OE Onoka CA Griffiths UK Cost-effectiveness analysis of rapid diagnostic test, microscopy and syndromic approach in the diagnosis of malaria in Nigeria: implications for scaling-up deployment of ACT Malar J 2009 8 265 10.1186/1475-2875-8-265 19930666 PMC2787522

[B37] Bell DJ Wootton D Mukaka M Montgomery J Kayange N Chimpeni P Hughes DA Molyneux ME Ward SA Winstanley PA Lalloo DG Measurement of adherence, drug concentrations and the effectiveness of artemether-lumefantrine, chlorproguanil-dapsone or sulphadoxine-pyrimethamine in the treatment of uncomplicated malaria in Malawi Malar J 2009 8 204 10.1186/1475-2875-8-204 19709418 PMC2744923

[B38] Piola P Fogg C Bajunirwe F Biraro S Grandesso F Ruzagira E Babigumira J Kigozi I Kiguli J Kyomuhendo J Ferradini L Taylor W Checchi F Guthmann J-P Supervised versus unsupervised intake of six-dose artemether-lumefantrine for treatment of acute, uncomplicated *Plasmodium falciparum *malaria in Mbarara, Uganda: a randomised trial Lancet 2005 365 1467 1473 10.1016/S0140-6736(05)66416-1 15850630

[B39] Republic of Kenya National guidelines for the diagnosis, treatment and prevention of malaria Book National guidelines for the diagnosis, treatment and prevention of malaria 2010 Nairobi

[B40] Harvey SA Jennings L Chinyama M Masaninga F Mulholland K Bell DR Improving community health worker use of malaria rapid diagnostic tests in Zambia: package instructions, job aid and job aid-plus-training Malar J 2008 7 160 10.1186/1475-2875-7-160 18718028 PMC2547110

[B41] Hawkes M Katsuva J Masumbuko C Use and limitations of malaria rapid diagnostic testing by community health workers in war-torn Democratic Republic of Congo Malar J 2009 8 308 10.1186/1475-2875-8-308 20028563 PMC2804690

[B42] Elmardi K Malik E Abdelgadir T Ali S Elsyed A Mudather M Elhassan A Adam I Feasibility and acceptability of home-based management of malaria strategy adapted to Sudan's conditions using artemisinin-based combination therapy and rapid diagnostic test Malar J 2009 8 39 10.1186/1475-2875-8-39 19272157 PMC2660358

[B43] Bundy D Shaeffer S Jukes M Beegle K Gillespie A Drake L Lee S-hF Hoffman A-M Jones J Mitchell A Barcelona D Camara B Golmar C Savioli L Sembene M Takeuchi T Wright C School-based Health and Nutrition Programs Disease Control Priorities in Developing Countries 2006

[B44] Fiedler J Chuko T The cost of Child Health Days: a case study of Ethiopia's Enhanced Outreach Strategy (EOS) Health Policy Plan 2008 23 222 10.1093/heapol/czn015 18562457

[B45] Mueller DH Wiseman V Bakusa D Morgah K Daré A Tchamdja P Cost-effectiveness analysis of insecticide-treated net distribution as part of the Togo Integrated Child Health Campaign Malar J 2008 7 73 10.1186/1475-2875-7-73 18445255 PMC2396647

[B46] O'meara WP Bejon P Mwangi TW Okiro EA Peshu N Snow RW Newton CRJC Marsh K Effect of a fall in malaria transmission on morbidity and mortality in Kilifi, Kenya Lancet 2008 372 1555 1562 10.1016/S0140-6736(08)61655-4 18984188 PMC2607008

[B47] Dicko A Diallo AI Tembine I Dicko Y Dara N Sidibe Y Santara G Diawara H Conaré T Djimde A Chandramohan D Cousens S Milligan PJ Diallo DA Doumbo OK Greenwood B Intermittent preventive treatment of malaria provides substantial protection against malaria in children already protected by an insecticide-treated bednet in mali: a randomised, double-blind, placebo-controlled trial PLoS Med 2011 8 e1000407 10.1371/journal.pmed.1000407 21304923 PMC3032550

[B48] Konaté AT Yaro JB Ouédraogo AZ Diarra A Gansané A Soulama I Kangoyé DT Kaboré Y Ouédraogo E Ouédraogo A Tiono AB Ouédraogo IN Chandramohan D Cousens S Milligan PJ Sirima SB Greenwood B Diallo DA Intermittent preventive treatment of malaria provides substantial protection against malaria in children already protected by an insecticide-treated bednet in burkina faso: a randomised, double-blind, placebo-controlled trial PLoS Med 2011 8 e1000408 10.1371/journal.pmed.1000408 21304925 PMC3032552

[B49] Le Port A Cot M Etard J-F Gaye O Migot-Nabias F Garcia A Relation between *Plasmodium falciparum *asymptomatic infection and malaria attacks in a cohort of Senegalese children Malar J 2008 7 193 10.1186/1475-2875-7-193 18823542 PMC2567330

[B50] Bassat Q Mulenga M Tinto H Piola P Borrmann S Menéndez C Nambozi M Valéa I Nabasumba C Sasi P Bacchieri A Corsi M Ubben D Talisuna A D'Alessandro U Dihydroartemisinin-piperaquine and artemether-lumefantrine for treating uncomplicated malaria in African children: a randomised, non-inferiority trial PLoS ONE 2009 4 e7871 10.1371/journal.pone.0007871 19936217 PMC2776302

[B51] Githeko AK Brandling-Bennett AD Beier M Atieli F Owaga M Collins FH The reservoir of *Plasmodium falciparum *malaria in a holoendemic area of western Kenya Trans R Soc Trop Med Hyg 1992 86 355 358 10.1016/0035-9203(92)90216-Y 1359683

[B52] Baliraine FN Afrane YA Amenya DA Bonizzoni M Menge DM Zhou G Zhong D Vardo-Zalik AM Githeko AK Yan G High prevalence of asymptomatic plasmodium falciparum infections in a highland area of western Kenya: a cohort study J INFECT DIS 2009 200 66 74 10.1086/599317 19476434 PMC2689925

[B53] Dal-Bianco MP Köster KB Kombila UD Kun JFJ Grobusch MP Ngoma GM Matsiegui PB Supan C Salazar CLO Missinou MA Issifou S Lell B Kremsner P High prevalence of asymptomatic *Plasmodium falciparum *infection in Gabonese adults Am J Trop Med Hyg 2007 77 939 942 17984357

[B54] Kern S Tiono A Makanga M Gbadoe AD Premji Z Gaye O Sagara I Ubben D Cousin M Oladiran F Sander O Ogutu B Community screening and treatment of asymptomatic carriers of *Plasmodium falciparum *with artemether-lumefantrine to reduce malaria disease burden: a modelling and simulation analysis Malar J 2011 10 210 10.1186/1475-2875-10-210 21801345 PMC3161019

[B55] Brooker S Kolaczinski JH Gitonga CW Noor AM Snow RW The use of schools for malaria surveillance and programme evaluation in Africa Malar J 2009 8 231 10.1186/1475-2875-8-231 19840372 PMC2768743

[B56] Yeung S Pongtavornpinyo W Hastings IM Mills AJ White NJ Antimalarial drug resistance, artemisinin-based combination therapy, and the contribution of modeling to elucidating policy choices Am J Trop Med Hyg 2004 71 179 186 15331836

[B57] Chuma J Okungu V Molyneux C The economic costs of malaria in four Kenyan districts: do household costs differ by disease endemicity? Malar J 2010 9 149 10.1186/1475-2875-9-149 20515508 PMC2890678

[B58] Coast J Is economic evaluation in touch with society's health values? BMJ 2004 329 1233 1236 10.1136/bmj.329.7476.1233 15550430 PMC529373

[B59] Williams I Bryan S Understanding the limited impact of economic evaluation in health care resource allocation: a conceptual framework Health Policy 2007 80 135 143 10.1016/j.healthpol.2006.03.006 16621124

[B60] Weatherly H Drummond M Claxton K Cookson R Ferguson B Godfrey C Rice N Sculpher M Sowden A Methods for assessing the cost-effectiveness of public health interventions: key challenges and recommendations Health Policy 2009 93 85 92 10.1016/j.healthpol.2009.07.012 19709773

[B61] Dicko A Sagara I Sissoko MS Guindo O Diallo AI Kone M Toure OB Sacko M Doumbo OK Impact of intermittent preventive treatment with sulphadoxine-pyrimethamine targeting the transmission season on the incidence of clinical malaria in children in Mali Malar J 2008 7 123 10.1186/1475-2875-7-123 18611271 PMC2500037

[B62] Ashton RA Kefyalew T Tesfaye G Pullan RL Yadeta D Reithinger R Kolaczinski JH Brooker S School-based surveys of malaria in Oromia Regional State, Ethiopia: a rapid survey method for malaria in low transmission settings Malar J 2011 10 25 10.1186/1475-2875-10-25 21288368 PMC3039636

[B63] Guyatt HL Brooker S Donnelly CA Can prevalence of infection in school-aged children be used as an index for assessing community prevalence? Parasitology 1999 118 Pt 3 257 268 10205801 10.1017/s0031182098003862

[B64] Mueller DH Abeku TA Okia M Rapuoda B Cox J Costs of early detection systems for epidemic malaria in highland areas of Kenya and Uganda Malar J 2009 8 17 10.1186/1475-2875-8-17 19149878 PMC2636824

[B65] O'Meara WP Smith DL McKenzie FE Potential impact of intermittent preventive treatment (IPT) on spread of drug-resistant malaria PLoS Med 2006 3 e141 10.1371/journal.pmed.0030141 16573365 PMC1440294

[B66] Pongtavornpinyo W Yeung S Hastings I Dondorp A Day N White N Spread of anti-malarial drug resistance: mathematical model with implications for ACT drug policies Malar J 2008 7 229 10.1186/1475-2875-7-229 18976503 PMC2585590

