# Supplementary material for: Cost analysis of school-based intermittent screening and treatment of malaria in Kenya
Source: Malar J. 2011 Sep 20;10:273. doi: 10.1186/1475-2875-10-273 (PMC3187739; doi:10.1186/1475-2875-10-273)
Supplement: Additional file 1 — Unit Costs. A list of ingredient unit costs and relevant data collected for this evaluation. [file 1475-2875-10-273-S1.PDF]

Supplementary Information

Unit Costs

**Item**	**Unit**	**Unit Cost KSH**	**Source**
**FIELD KIT**			
Parachecks	Test	105.84	KEMRI-Wellcome
Coartem	Course 5 - 15kg	24.7628	DOMC
	Course 15 - 25kg	49.5256	DOMC
	Course 25 - 35kg	72.6908	DOMC
	Course >35kg	98.2524	DOMC
Blood lancets	Box of 100	280	KEMRI-Wellcome
Cotton Wool	Box	127.5	KEMRI-Wellcome
Gauze Roll	Roll	452	KEMRI-Wellcome
Gloves	Box of 50 pairs	300	KEMRI-Wellcome
Paper towels	Roll	60	KEMRI-Wellcome
Dettol dispenser	Item	300	KEMRI-Wellcome
Thermometer	Item	550	KEMRI-Wellcome
Biscuit packs	Box of 60	275	KEMRI-Wellcome
Milk cartons	Item	27	KEMRI-Wellcome
Water (asilia)	Bottle	20	KEMRI-Wellcome
Paracetamol	Bottle of 1000	350	KEMRI-Wellcome
Pencils	Item	30	KEMRI-Wellcome
Erasers	Item	12	KEMRI-Wellcome
Sharpeners	Item	10	KEMRI-Wellcome
Masking tape	Item	80	KEMRI-Wellcome
Garbage bag	Roll of 25	524	KEMRI-Wellcome
Marker pens	Item	80	KEMRI-Wellcome
Scissors	Item	150	KEMRI-Wellcome
Dust bin	Item	150	KEMRI-Wellcome
Triple timers	Item	1464	KEMRI-Wellcome
Weighing scales	Item	1600	KEMRI-Wellcome
**FACILITY**			
Rent	per month	27000	KEMRI-Wellcome
Internet	per month	2500	KEMRI-Wellcome
mail and courier	per month	2573	KEMRI-Wellcome
telephone credit	per day	750	KEMRI-Wellcome
Ink	Cartidge	6000	KEMRI-Wellcome
Paper	Box of 2500	2100	KEMRI-Wellcome
Pens	Item	80	KEMRI-Wellcome
Personal computer	Item	70000	KEMRI-Wellcome
Software (MS office)	Item	4000	http://www.pcworld.co.ke
Printer	Item	17500	KEMRI-Wellcome
Photocopying	per page	2	KEMRI-Wellcome

Unit Costs

Item	Unit	Unit Cost KSH	Source
**TRANSPORT**			
Ford Ranger	Purchase	2458152	Daily Nation advertisement
Fuel	Litre	88.37	Oberved
Insurance	per year	246143	AMACO insurance company
Maintanence	every 5000km	17500	KEMRI-Wellcome
Distribution: Nairobi to Msambweni	per box	138	Keny Medical Supplies Agency (KEMSA)
**PERSONNEL (pay grades)**			
Nurse (J, K)	per month	50284	MOH band mid point
Technologist (J, K)	per month	50284	MOH band mid point
Driver (G, H)	per month	22131	MOH band mid point
Co-ordinator (N)	per month	85975	MOH band mid point
Logistics (J,K)	per month	50284	MOH band mid point
IT support (J,K)	per month	50284	MOH band mid point
Purchasing (J,K)	per month	50284	MOH band mid point
Clinical Officer (N)	per month	85975	MOH band mid point
Pharmacist (M)	per month	70086	MOH band mid point
Training Officer (J, K)	per month	50284	MOH band mid point
Teacher	per month	23733	DAILY NATION
Community Worker	per day	1200	KEMRI

Sensitivity alternatives

RDT			
CareStart Malaria Pf/Pv Combo	Per Test	61.61	Carramore International
First Response Malaria Ag HRP2	Per Test	48.46	Carramore International
Advantage Pf Malaria Card	Per Test	80.23	Carramore International
SD Bioline Malaria Pan/Pf	Per Test	132.15	Carramore International
NOW Malaria Test Kit	Per Test	263.06	Carramore International
Antimalarial			
Dihydroartemisinin-Piperaquine		59.15	http://erc.msh.org
Sulphadoxine-Pyrimethamine		9.96	http://erc.msh.org

Kenyan CPI Inflation Rates (Index Mundi)

2005	2006	2008	2009
9	10.3	9.7	26.3
